# HUS1 as a Potential Therapeutic Target in Urothelial Cancer

**DOI:** 10.3390/jcm11082208

**Published:** 2022-04-15

**Authors:** Andrea Katharina Lindner, Tobias Furlan, Jacob J. Orme, Gennadi Tulchiner, Nina Staudacher, David D’Andrea, Zoran Culig, Renate Pichler

**Affiliations:** 1 Department of Urology, Medical University of Innsbruck, Anichstrasse 35, 6020 Innsbruck, Austria; andrea.lindner@i-med.ac.at (A.K.L.); tobias.furlan@i-med.ac.at (T.F.); gennadi.tulchiner@i-med.ac.at (G.T.); nina.staudacher@i-med.ac.at (N.S.); 2 Division of Medical Oncology, Mayo Clinic, First Street SW, Rochester, MN 55905, USA; orme.jacob@mayo.edu; 3 Department of Urology, Medical University of Vienna, Währinger Gürtel 18-20, 1090 Wien, Austria; david.dandrea@meduniwien.ac.at

**Keywords:** bladder cancer, prognosis, biomarker, chemotherapy, platinum-based, HUS1, resistance, DNA damage repair, 9-1-1 complex

## Abstract

Platinum-based chemotherapy is the standard of care with concern to first-line systemic therapy for metastatic disease in urothelial cancer (UC). Resistance to chemotherapy despite an initial response is linked with the ability to remove platinum-based DNA adducts and to repair chemotherapy-induced DNA lesions by various DNA repair proteins. The Rad9-Rad1-HUS1 complex that is loaded onto DNA at sites of damage is involved in checkpoint activation as well as DNA repair. Here, we addressed for the first time the potential influence of HUS1 expression in urothelial carcinogenesis (using two human basal urothelial cancer cell lines UM-UC-3 and HT1197) and its role as a potential therapeutic target for predicting responses to platinum-based chemotherapy. Specific inhibition of HUS1 expression in both cell lines was achieved by specific siRNA and validated by Western blot. In order to define the possible importance of HUS1 in the regulation of cellular proliferation, parental and resistant cells were treated with increasing concentrations of either control or HUS1 siRNA. HUS1 protein expression was observed in both human basal urothelial cancer cell lines UM-UC-3 and HT1197. In cisplatin-sensitive cells, knock-down of HUS1 inhibited cellular proliferation in the presence of cisplatin. On the contrary, knock-down of HUS1 in resistant cells did not result in a re-sensitization to cisplatin. Finally, RNAseq data from the Cancer Genome Atlas provided evidence that HUS1 expression is a significant prognostic factor for poor survival in UC patients. In summary, HUS1 may acts as an oncogene in UC and might be a key determinant of the cellular response to cisplatin-based chemotherapy.

## 1. Introduction

In the locally advanced setting of muscle-invasive bladder cancer, neoadjuvant cisplatin-based chemotherapy (NAC) followed by radical cystectomy (RC) is still the standard of care in cisplatin-fit patients according to the current EAU guidelines [1]. Various clinical trials testing immunotherapy or targeted therapies (as monotherapies or in combination with chemotherapy) in the neoadjuvant setting are ongoing, with promising results. The rate of pathologically free of disease (pT0) occurrence with RC alone is low (15%), whereas a substantial proportion of patients (38%) will be rendered pathologically free of cancer (pT0) at the time of RC after undergoing NAC [2]. Additionally, NAC improves overall survival (OS; 5–8% at five years) [3].

Moreover, nearly half of patients undergoing RC are cisplatin-ineligible to receive NAC based on poor renal function status [4]. Finally, the utilization of NAC in clinical practice is low [5,6]. Possible causes of the low clinical acceptance rate for the application of NAC are a time delay of RC in NAC non-responders, the potential chemotherapy-induced toxicity rate, and the lack of predictive, clinically applicable biomarkers for identifying patients most likely to benefit from NAC [7].

The resistance of cancer cells to cisplatin is mostly based on the ability to remove cisplatin-DNA adducts and to repair cisplatin-induced DNA lesions by the presence of certain DNA repair proteins [8]. It has been shown that DNA-damage repair APOBEC genetic alterations drive the most common mutations in muscle-invasive bladder cancer (MIBC) [9,10], resulting in a better response to NAC via genomic alterations in DNA damage response (DDR) genes [11]. To validate potential predictive biomarkers of response to NAC, we recently performed a comprehensive biomarker analysis of primary MIBC specimens prior to NAC on a genetic and molecular level to characterize driver mutations, chromosomal somatic changes, genome-wide frequency of copy number alterations, and mutational signatures [12]. For the first time, assessing somatic copy number alterations by Affymetrix arrays, we detected a specific amplification on the chromosomal region *7p12* associated with non-response to NAC and, consequently, worse survival outcomes after RC. When analyzing the sequence of interest in detail, *7p12.2–p11.2* prioritized genes that had not previously been reported as regulators of platinum-based chemotherapy responses in bladder cancer including HUS1, *ABCA13*, *IKZF1*, *EGFR*, and *FIGNL1* [12]. HUS1, a member of the multifaceted DDR network for maintaining genomic integrity, is involved in cell cycle arrest and DNA repair in response to DNA damage and regulates the response to genotoxic chemotherapies in vivo [13,14]. In response to DNA damage, HUS1 is involved in a complex with two other proteins such as Rad1 and Rad 9, known as the 9-1-1 complex, an element of the DNA damage checkpoint response that regulates cell cycle arrest at the G2 checkpoint with Chk1 activation [15]. In addition to its role in checkpoint activation, accumulating evidence suggests that the Rad9-Rad1-HUS1 complex also participates in DNA repair. In summary, the Rad9-Rad1-HUS1-complex that is loaded onto DNA at sites of damage is involved in checkpoint activation and DNA repair [16]. Previously, it was demonstrated that non-small lung cancer cells treated with HUS1 antisense oligonucleotides increase their sensitivity to cisplatin [17]. Moreover, loss of HUS1 sensitized cells to etoposid-induced apoptosis [18]. However, its role in bladder cancer development, progression, and chemosensitivity has not yet been studied.

The aim of this translational oncology study is to elucidate in detail, for the first time, the role of human HUS1 in urothelial carcinogenesis and its role as a potential therapeutic target for predicting response to platinum-based chemotherapy.

## 2. Materials and Methods

### 2.1. Cell Culture

UM-UC-3 cells were purchased from Sigma Aldrich (96020936, St. Louis, MO, USA) and cultured in DMEM (Lonza, Basel, Switzerland) with 10% fetal bovine serum (PAN Biotech, Aidenbach, Germany), 1× penicillin/streptomycin (Lonza, Basel, Switzerland) and 1× GlutaMAX (Lonza, Basel, Switzerland). Cisplatin-resistant cells were generated by chronic treatment with increasing concentrations of Cisplatin, resulting in UM-UC-3 CisR cells, which are viable in growth medium containing 2 µM Cisplatin.

### 2.2. HUS1 Knockdown

ON-TARGET plus SMARTpool Human HUS1 siRNA (THP) or ON-TARGETplus siControl SMARTPool (THP) and Lipofectamine RNAiMAX (Thermo Fisher, Waltham, MA, USA) were used according to the manual. In short: 9 µL Lipofectamine RNAiMAX and 30 pmol siRNA per well were diluted in 150 µL Opti-MEM (Gibco, Thermo Fisher) each and mixed. The mixture was then incubated for 5 min and added to 2 mL cell suspension (500,000 cells/well) in a 6-well plate. Cells were incubated overnight and the growth medium was changed. After 3 days, cells were either lysed using radio-immunoprecipitation assay buffer or used for viability assays.

### 2.3. Western Blot

Electrophoretic separation was performed by loading 20 mg protein into 3 to 8% Tris-Acetate protein gels (Thermo Fisher, Waltham, MA, USA). For blotting, a 0.2 µm Amersham Protran Nitrocellulose membrane (Sigma, St. Louis, MO, USA) was used. Revert 700 Total Protein Stain (LI-COR) was then used to quantify total protein according to the manual. Membranes were blocked in Starting Blocking Buffer (Thermo Fisher, Waltham, MA, USA) for 1 h at room temperature. HUS1 primary antibody (Abcam, ab96297, Cambridge, UK) was incubated overnight at 4 °C. The membranes were washed with Tris-buffered saline containing 0.1% Tween-20 and incubated with IRDye Goat anti-Rabbit IgG Secondary Antibody (LI-COR Bio-sciences) for 45 min. Membranes were scanned using the Odyssey imaging System (LI-COR). Image Studio software (v5.2, LI-COR) was used to quantify protein ratios.

### 2.4. Viability Assay

450 cells per well were seeded in a 384 well-plate (Corning, New York, NY, USA) in triplicate. Cells were treated with different Cisplatin concentrations for 96 h. After 24 h, the reagents of the RealTime-Glo MT Cell viability assay (Promega, Madison, WI, USA) were added as described by the manual and quantified on a Cytation5 (BioTek, Winooski, VT, USA) plate reader. GraphPad Prism 8 was used for visualization.

### 2.5. Cancer Genome Atlas Data

Level 3 data including sequence per million mapped fragments (FPKM) transcript data from validated RNA-seq experiments was downloaded for all available patients with chemotherapy-naïve high-grade urothelial cancer in The Cancer Genome Atlas (TCGA) database, as previously published [19]. Survival analysis was performed on OS by Cox proportional hazards modeling and all data were right-censored for analysis. Cutoffs were generated by iterative Cox proportional hazards modeling without correction for age or stage of tumor, as described [20]. *p* < 0.05 was considered statistically significant.

## 3. Results

### 3.1. Expression of HUS-1 in Urothelial Cancer Cell Lines

**Figure 1:**
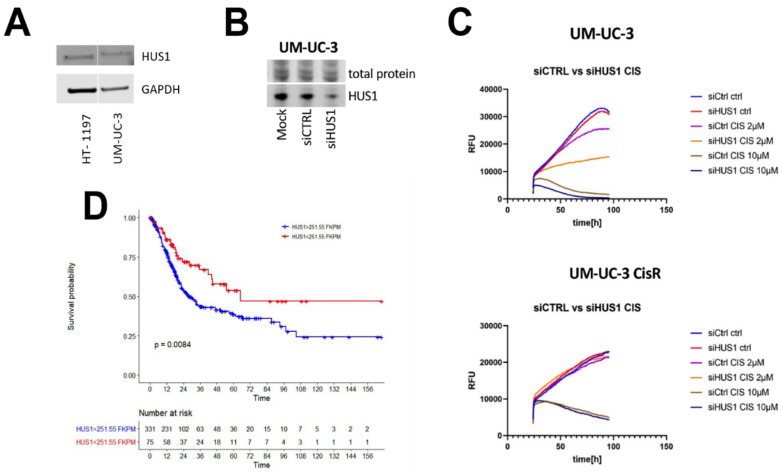
HUS1 is expressed in urothelial cancer cell line UM-UC-3 but has differential effects on cellular viability after treatment with cisplatin. (**A**) HUS1 protein expression in human basal urothelial cancer cell lines UM-UC-3 and HT1197. (**B**) HUS1 expression was determined by Western Blot in UM-UC-3 cells treated with either specific or control siRNA. (**C**) Response of UM-UC-3 cells and their cisplatin-resistant derivative to cisplatin after treatment with either control or HUS1 siRNA. (**D**) Kaplan–Meier survival analysis concerning HUS1 mRNA expression in bladder cancer extracted from TGCA database. High mRNA expression of HUS1 is significantly associated with inferior survival compared with low mRNA expression levels.

**Figure A1:**
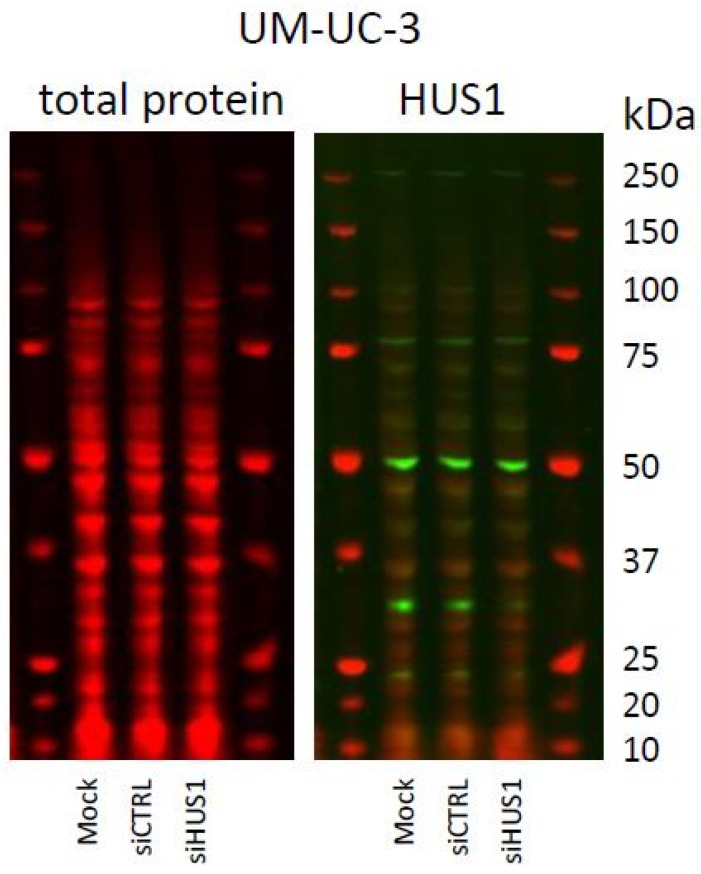
Whole blot relating to Figure 1A.

Initially, we sought to establish the role of HUS1 in urothelial carcinogenesis and therapy response in our research. HUS1 protein expression was observed in human basal urothelial cancer cell lines UM-UC-3 and HT1197 (results shown in Figure 1A,B and Appendix A Figure A1).

Functional consequences of HUS1 down-regulation were investigated in both cell lines and their cisplatin-resistant derivatives. Specific inhibition of HUS1 down-regulation were investigated in both cell lines and their cisplatin-resistant derivatives. Specific inhibition of HUS1 expression in both cell lines was achieved by specific siRNA and validated by Western blot (Figure 1B and Figure A1).

In order to define the possible importance of HUS1 in the regulation of cellular proliferation, we treated parental and resistant cells with increasing concentrations of either control or HUS1 siRNA. Most interestingly, our data show for the first time that, in cisplatin-sensitive cells, knock-down of HUS1 inhibits cellular proliferation in the presence of cisplatin (Figure 1C). At cisplatin concentrations higher than 2 µM, no difference was observed. In contrast, knock-down of HUS1 in resistant cells did not lead to re-sensitization to cisplatin.

### 3.2. Prognostic Significance of HUS1 Expression in Patients with Bladder Cancer

In line with our preliminary results, TCGA (The Cancer Genome Atlas) database provides the first evidence that mRNA expression of HUS1 is a statistically significant prognostic parameter of poor survival in bladder cancer patients (Figure 1D).

## 4. Discussion

The 9-1-1 complex facilitates the ATR-mediated phosphorylation and activation of CHK1, a protein kinase that regulates S-phase progression, G2/M arrest, and replication fork stabilization. Thus, the 9-1-1 complex serves a dual role as a DNA-damage sensor in checkpoint signaling and as a mediator in the DNA repair pathway [21]. Independently of ATR activation, the 9-1-1 complex can also directly interact with members of homologous recombination or mismatch repair, base excision repair factors and translesion synthesis polymerases [22,23,24]. There is growing evidence that ATR, CHK1 and the 9-1-1 complex are important drivers for tumor progression [25,26,27]. However, how pathway-specific DDR inhibition could increase the efficacy and reduce the toxicity of chemotherapy is still unclear. In vivo data argue that a targeted inhibition of the 9-1-1 complex such as with HUS1 may be an effective strategy for the treatment of ATM-deficient and other cancers [13]. Moreover, inactivation of HUS1 or ATR in mice is synergized with p53 loss, inducing apoptosis but not tumorigenesis [28,29].

In our paper, we provide the first evidence that HUS1 acts as an oncogene in urothelial cancer. This is different to previous results in hepatocellular carcinoma [14] in which HUS1 was demonstrated to act as a tumor suppressor, as evidenced in experiments assessing cell proliferation and colony formation assays and migration and invasion assays. Its expression was downregulated in hepatocellular carcinoma cells and human samples. On the other hand, HUS1, when acting as an oncogene, may be a target for tumor-suppressive miRNA. Such a situation has been described in lung cancer, in which HUS1 is targeted by MiR-340-3p [30]. It was shown that, in lung cancer, HUS1 promotes the ability of cells to proliferate or migrate. Kinzel et al., provided the first evidence that the specific inhibition of HUS1 enhances cisplatin sensitivity in human H1299 lung cancer cells [17]. In addition, HUS1 expression was defined as a poor prognostic parameter for ovarian cancer [26]. In chemo-resistant breast cancers, Rad9 had a high expression, with no significant variations in Rad9 expression levels between pre- and post-chemotherapeutic tumor specimens. This fact indicates that Rad9-Rad1-HUS1 overexpression may contribute to an innate resistance of tumor cell responses to chemotherapy [31].

HUS1 seems to be a key determinant of the cellular response to cisplatin-based chemotherapy, as knock-down of HUS1 inhibited cellular proliferation in the presence of cisplatin. Thus, an early-HUS1 therapeutic intervention might be advantageous in order to improve the therapeutic efficacy of platinum-based chemotherapy. RNA interference has been used to inhibit RAD9 in prostate cancer [32]. This treatment leads to enhanced radiation sensitivity, which was explained by the inhibition of integrin ß1. Such experimental approaches may represent the first step in the development of innovative bladder cancer therapies.

Additionally, DDR mechanisms are not only essential for responses to chemotherapy, but also regulate the programmed death-ligand (PD-L1) expression on tumor cells or immune cells by activating cell cycle checkpoints, producing neo-antigen epitopes, and other mechanisms which may be a potential therapeutic target for immunotherapy. PD-L1 inhibition has been established in therapies for localized bladder cancer [33]. However, more research is needed in order to further improve anti-PD-L1 combination therapies with radiation or targeted therapies. Therefore, further understanding how the RAD9-RAD1-HUS1 complex affects immune checkpoints in bladder cancer cells is also of particular interest.

In contrast, our data also provided evidences that cisplatin-resistant cells, which do not express HUS1, cannot be inhibited by cisplatin. This finding is of particular interest. In order to analyze the possible reasons for this, an improved understanding and analyses of the inter-dependency between the 9-1-1 cell-cycle checkpoint response complex are needed. It is hypothesized that the loss of HUS1 leads to a destabilization of the 9-1-1 complex. To further investigate this hypothesis, immunofluorescent staining of Rad1 and Rad9 in cells treated with HUS1 siRNA will be the next step in our research.

Since several novel targeted therapies are being developed for metastatic bladder cancer patients, refractory to platinum-based chemotherapy and immunotherapy—including FGFR inhibitors [34] as well as antibody-drug conjugates [35]—future studies may be important for clarifying the role of HUS1 in the regulation of DNA repair in this malignancy. FGFR3 alterations are particularly important as they can be detected in about 18% of all urothelial carcinoma cases [34].

In summary, in addition to our previous study, we show for the first time that HUS1 likely acts as an oncogene in urothelial cancer. Its interaction with currently approved and experimental drugs will be a subject of future research. This research may be facilitated by an increasing number of pre-clinical models including organoids, conditionally reprogrammed cell cultures, genetically engineered mouse models, and patient-derived xenografts. Thinking ahead, it is of great importance that our findings can be implemented and supportive towards further research in future settings of human analyses. Possible promising steps should include the validation of HUS1 expression from tissue blocks acquired after transurethral resection. Additionally, large cohort retrospective studies concentrating on gene expression in correlation to therapy response and platin-resistance would be further steps towards supporting the role of HUS1 as a key determinant in UC.

## Data Availability

The data presented in this study are available on request from the corresponding author.
